# Medicine in China

**Published:** 1886-07

**Authors:** 


### MEDICINE IN CHINA.

Lucy Stritmatter, writing on the practice of medicine in China, in *Columbus Medical Journal*, says, that in China every man and woman is a dyspeptic, there are causes apparent to any critical observer. For instance, the people live principally on rice and vegetables. The rice is steamed just enough to warm it through ; the vegetables are simply wilted. In this uncooked condition they are eaten in enormous quantities, the meal coming only twice a day. Can you wonder they are dyspeptics ?

As to paralysis, which is common, there are two theories. One is that it is due to lead poisoning, as the people drink largely from leaden vessels. The other, that it is due to insufficient nourishment. This last I believe to be true. I always treated may cases with beef extract, mutton and chicken broth, tonics and electricity, and I found it a very successful treatment.

It is well known that for centuries a system of medicine has been practiced in China, if system it can be called. I speak of it as a system, because I have found it so referred to in both English and Chinese literature. What this practice is I wish to show you, but before I pass to the doctors, let me dwell a moment on Chinese pharmacy. Gentian is imported from Corea in such quantities that it furnishes a staple of national commerce. The mountainous parts of their own country furnish an endless variety of herbs, which are used generally root, branch and leaf. Many of our most powerful drugs, such as mercury, digitalis, etc., are found in crude forms in their shops.

When a child is born, the umbilical cord is measured to the top of the child's head, cut from the placenta at that place, and when this mass of decaying cord loosens from the child it is sold to the druggist. Cat's heads, and many other disgusting things, are purchased by the same functionary. The body of a still-born child, provided said child is a first born and a son, is supposed to contain medicinal properties.

With such a pharmacopeia, what can we expect in the profession? We find exactly this: A set of ignorant, superstitious men, who do great harm, and if they do any good it is purely accidental.

I give below the translation of a prescription, which was written by a native doctor for a man who had applied to him with chronic dysentery:

Powdered gentian;
" catnip leaves;
" root of taraxicum;
" root of white ginger;
" black pepper;
" smilax;
" root of persimmon tree;
Of each quantity sufficient to half fill a wine glass;
Powdered rhubarb;
" diftalis.

Calomel,
Of each as much as can be held between the thumb and forefinger.
Three inches of an umbilical cord, powdered;
One snake skin, powdered;
One cat's head, fresh if possible.

Sig. Boil all these in five quarts of water for three hours; cover the patient with many quilts, and cause him to drink all the medicine within thirty-six hours, as hot as it can be swallowed.

I have found prescriptions very near like this one in the hands of patients suffering from leprosy, pneumonia, phthisis, small-pox, paralysis, typhoid fever, etc., proving, as I believe, that their treatment is entirely haphazard.

Gynecology and obstetrics are not practiced at all by the doctors, the seclusion in which the women live in that land rendering it impossible.

Surgery is practiced in the same barbarous and ignorant manner which we have noticed in their general therapeutics. I will give one instance which fell under my observation. I saw a surgeon drive three steel spikes, about a finger in length, into a rheumatic knee. Of course the result was an anchylosed joint. The patient endured the operation with great fortitude, having no anesthetic.

I found the people of the country more susceptible to my remedies than the patients in my English-speaking practice. The faith of the natives in foreign doctors and their remedies is unlimited, and for eight years I carried on a pleasant and successful, though very laborious, practice among them.

I say I found them unusually susceptible to the action of our methods of medication, but that is not true in cases where opium was indicated, owing to the fact that the people are to so large an extent, addicted to the daily use of that drug.

